# Evolutionary change in flight-to-light response in urban moths comes with changes in wing morphology

**DOI:** 10.1098/rsbl.2023.0486

**Published:** 2024-03-13

**Authors:** Evert Van de Schoot, Thomas Merckx, Dieter Ebert, Renate A. Wesselingh, Florian Altermatt, Hans Van Dyck

**Affiliations:** ^1^ Earth & Life Institute, UCLouvain, Louvain-la-Neuve 1348, Belgium; ^2^ WILD, Biology Department, Vrije Universiteit Brussel, Brussels 1050, Belgium; ^3^ Department of Environmental Sciences, Zoology, University of Basel, Basel, Switzerland; ^4^ Department of Aquatic Ecology, Eawag, Swiss Federal Institute of Aquatic Science and Technology, Dübendorf, Switzerland; ^5^ Department of Evolutionary Biology and Environmental Studies, University of Zurich, Zurich, Switzerland

**Keywords:** flight-to-light behaviour, urban ecology, anthropogenic change, wing morphology, artificial light at night (ALAN), urban evolution

## Abstract

Moths and other insects are attracted by artificial light sources. This flight-to-light behaviour disrupts their general activity focused on finding resources, such as mating partners, and increases predation risk. It thus has substantial fitness costs. In illuminated urban areas, spindle ermine moths *Yponomeuta cagnagella* were reported to have evolved a reduced flight-to-light response. Yet, the specific mechanism remained unknown, and was hypothesized to involve either changes in visual perception or general flight ability or overall mobility traits. Here, we test whether spindle ermine moths from urban and rural populations—with known differences in flight-to-light responses—differ in flight-related morphological traits. Urban individuals were found to have on average smaller wings than rural moths, which in turn correlated with a lower probability of being attracted to an artificial light source. Our finding supports the reduced mobility hypothesis, which states that reduced mobility in urban areas is associated with specific morphological changes in the flight apparatus.

## 1. Introduction

Ongoing urbanization is a textbook example of the ever-increasing human impact on the environment. The United Nations [1] predicts that more than two-thirds of the world's population will live in urban areas by 2050, driving strong expansion of the urban land area [2]. For many species, urbanization goes hand in hand with a deterioration of environmental quality caused by diverse processes, one of them being light pollution. Since the review by Longcore & Rich in 2004 [3], the interest for the consequences of light pollution—caused by artificial light at night (ALAN)—has strongly increased, particularly for its effects on nocturnal insects [4–9]. ALAN mainly affects insects in two ways. Firstly, it alters day–night cycles, disrupting life cycle regulation (e.g. disrupted diapause induction) [10–12] and changing plant–pollinator interactions [13–15]. Secondly, many insects experience negative consequences from their innate positive or negative phototaxis, causing them to be attracted [16] or repelled [17] by ALAN. This, in turn, leads insects away from their favourable habitats [18] and can result in higher predation risk. Nocturnal insects are also distracted or restrained from essential behaviours like foraging [19] and reproduction [20]. These changes do not only result in ecological alteration, but may also operate as selective agents potentially driving rapid evolution, which may lead to phenotypic divergence between urban and rural populations [21,22]. In insects, and especially Lepidoptera, selection on flight performance is usually associated with changes in morphology, including body mass, thorax mass (containing the flight muscles), wing size, wing shape and wing loading [23–25]. Merckx *et al*. [26] showed intraspecific shifts towards larger and thus more mobile individuals in urban macro-moth populations. However, their study did not specifically test for the effect of ALAN. Furthermore, selection on flight behaviour stemming from changes in light environments might differ across species [27].

Altermatt & Ebert [28] showed experimentally that spindle ermine moths (*Yponomeuta cagnagella*) from urban populations showed a reduced attraction to light compared to individuals from rural populations. Moths originating from light-polluted urban areas, although reared under common-garden settings, had a 30% weaker flight-to-light response than conspecifics from pristine dark areas. These results suggest evolution of this trait in urban environments, reducing an ALAN-mediated ecological trap effect. Altermatt & Ebert [28] discussed two mutually non-exclusive mechanisms explaining the observed patterns: first, a decreased perception of light (see also [16]); and second, a reduced overall mobility in urban moths, which could also result from a (non-adaptive) by-product of selection on other traits. Here, we used the specimens from this previous study to investigate changes in flight-related morphology of ermine moths in urban areas and test for their propensity to flight to the light.

## 2. Material and methods

### (a) Study species and sampling sites

We used adults of the spindle ermine moth *Yponomeuta cagnagella* (Hübner) (Lepidoptera: Yponomeutidae) to study differences in flight-related morphological traits between individuals from rural versus urban populations, reared under common-garden settings. This small moth species is common in Europe and West Asia. Adults fly from the end of June to October. Caterpillars of *Y. cagnagella* are the only species in the *Yponomeuta* species complex to feed monophagously on spindle trees (*Euonymus* spp.) and especially *Euonymus europaeus* L. is frequently used as a host plant. We analysed specimens used in the experiment of Altermatt & Ebert [28], which were stored at −20°C after the flight-to-light experiment.

In the original experiment, second-instar larvae were collected on *Euonymus europaeus* in 11 populations in Switzerland and France. Five populations were located in urbanized areas with high levels of ALAN (radiance (L) > 40 10^−9^ Wm^−2^ sr^−1^) while six populations were located in dark, rural areas (L < 3 × 10^−9^ Wm^−2^ sr^−1^) (electronic supplementary material, figure S1). For each population, larvae from one to six full-sib families were reared under common-garden conditions in the lab. Two to 3 days after eclosion, adult moths were individually colour-marked on the wings and their flight-to-light response was tested. Moths were released at one side of a walk-in cage (bottom: 5.7 × 2.5 m, top: 5.7 × 1.8 m, height: 3 m) with a Heath actinic (6 W) light trap at the opposite end. Eight hours after release, the light trap was checked for captured moths. Both captured and non-captured moths were counted, collected and stored in a freezer (−20°C) (see [28] for more details). Morphological analyses were done at UCLouvain (*N* = 680, a few individuals could no longer be identified due to loss of colour marking).

### (b) Flight-related morphology

#### (i) Thorax and abdomen mass

The moths were placed in an incubator at 60°C for 4 h. Preliminary tests had shown that dry body mass reached stable values after this period. Because (parts of) antennae and legs were often lacking from dried specimens, antennae and legs were systematically removed from all individuals before measuring total dry body mass (*m*_tot_) with a microbalance (Mettler MT5; precision ± 0.001 mg). We separated head, thorax, abdomen and wings with needle and tweezers and determined the thorax (*m*_th_) and abdomen mass (*m*_ab_). Relative thorax (*m*_th,rel_) and abdomen mass (*m*_ab,rel_) were calculated by dividing by total dry body mass.

#### (ii) Wing morphology

**Figure 1.**
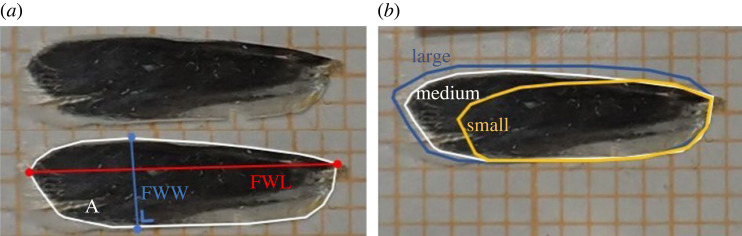
(*a*) *Yponomeuta cagnagella* forewing indicating the three morphological measurements taken: area (A), forewing length (FWL) and forewing width (FWW). (*b*) Contours of medium-sized forewing (white) with contours of relatively large (blue) and small (yellow) wings, respectively. Orange grid lines in the background indicate a millimetre scale.

We took standardized pictures (Olympus TG-6 camera) of the ventral side (where a clear difference between wing surface and fringe is visible) of the right forewing (figure 1). In the case of significant wing damage, the left forewing was used instead. Forewing length (*FWL*) (from the far left to right point of the dark grey zone), forewing width (*FWW*) (from the outermost (highest) point of the costa in a line perpendicular to the dorsal edge) and forewing area (*A*) were measured using ImageJ software (https://imagej.net/ij/index.html) on size-calibrated pictures. Length and width were measured twice and the mean value was used in the analyses. From these wing measures, we calculated aspect ratio (*AR*) as 4 · FWL²/FWW and wing loading (*WL*) as m_tot_/A [23,29]. The measurements were made without knowing the origin of the animals.

### (c) Statistical analysis

We tested for differences in morphological traits between samples from light polluted (urban) and dark (rural) population origin, using linear mixed effect regression models. Analyses were done separately for both sexes, since there was strong collinearity between the factors ‘sex’ and ‘body mass’ (females are heavier than males), body mass being also allometrically correlated with several of the morphological traits. For the analysis of the relationship between flight-to-light response and morphology, generalized linear mixed models with a binomial error distribution were used. Total body mass (*m*_tot_) was included as a covariate, except in models with *m_th_*_,rel_, *m_ab_*_,rel_ and *WL*. Family identity nested within population identity was used as a random factor in all models. All analyses were performed in R 4.2.2 [30]. We adopted the language of evidence in the results section [31]. Raw means and SE's are reported in the electronic supplementary material (electronic supplementary material, table S1 and figure S2).

## 3. Results

**Figure 2.**
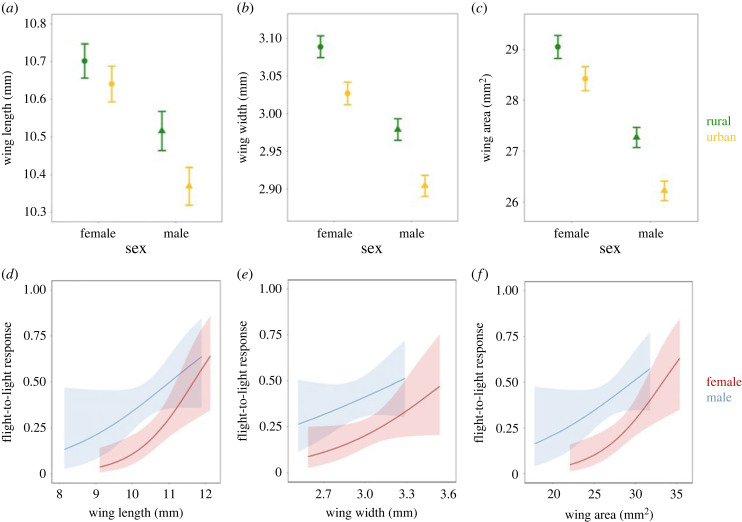
(*a–c*) Predicted values ± SE of wing length, wing width and wing area in female and male spindle ermine moths (*Yponomeuta cagnagella*) from rural and urban populations. (*d–f*) Predicted effects and 95% confidence intervals (shaded area) of wing length, wing width and wing area on flight-to-light response for females and males.

We have strong evidence that female moths from light-polluted, urban origins had narrower wings (on average by 2%) and higher aspect ratio (by 1.5%) than rural-origin females from dark night conditions (table 1, figure 2*a,b*). Urban females also had smaller wing areas compared to rural females, but with weaker evidence (table 1, figure 2*c*). Relative abdomen mass was on average 2.5% lower in urban females, again with weaker evidence, while total body mass, relative thorax mass and wing loading showed no significant differences (table 1).

**Table 1.** Differences in flight-related morphology between rural and urban spindle ermine moths. For each morphological trait the estimated mean ± SE (*n*) is given. All tests are based on d.f. = 1. Significant *p*-values (< 0.05) are given in bold, whereas statistical trend values reflecting weaker evidence (< 0.1) are indicated with a ° symbol.

	female				male			
	rural	urban	Wald *χ*²	*p*	rural	urban	Wald *χ*²	*p*
total body mass (mg)	12.871 ± 0.445 (182)	13.362 ± 0.473 (159)	0.57	0.450	7.674 ± 0.251 (147)	8.329 ± 0.258 (192)	3.31	0.069°
relative thorax mass	0.214 ± 0.005 (182)	0.222 ± 0.005 (159)	1.23	0.267	0.269 ± 0.006 (147)	0.262 ± 0.006 (190)	0.872	0.350
relative abdomen mass	0.697 ± 0.007 (182)	0.680 ± 0.007 (159)	3.16	0.076°	0.605 ± 0.007 (147)	0.614 ± 0.007 (192)	0.74	0.390
wing length (mm)	10.702 ± 0.045 (176)	10.641 ± 0.048 (143)	0.86	0.354	10.516 ± 0.052 (138)	10.369 ± 0.050 (180)	4.11	**0.043**
wing width (mm)	3.089 ± 0.014 (181)	3.027 ± 0.015 (157)	8.88	**0**.**003**	2.979 ± 0.014 (147)	2.904 ± 0.014 (191)	13.80	**< 0**.**001**
wing area (mm²)	29.047 ± 0.225 (179)	28.422 ± 0.236 (146)	3.66	0.056°	27.269 ± 0.199 (140)	26.220 ± 0.192 (182)	14.19	**< 0**.**001**
aspect ratio	15.764 ± 0.072 (176)	15.998 ± 0.077 (141)	4.93	**0**.**026**	16.243 ± 0.092 (138)	16.422 ± 0.089 (179)	1.91	0.167
wing loading (mg mm^−2^)	0.446 ± 0.011 (179)	0.466 ± 0.011 (146)	1.69	0.194	0.285 ± 0.007 (140)	0.315 ± 0.007 (182)	10.00	**0**.**002**

We tested whether the morphological traits are related to the flight-to-light response. Females with shorter, narrower and smaller wings and higher wing loading showed a weaker flight-to-light response (strong evidence) (table 2, figure 2*d–f*), especially when they originated from urban populations (Wald *χ*^2^ = 6.17, *p* = 0.013); in rural-origin females the same relationship was found, but weaker (Wald *χ*^2^ = 2.90, *p* = 0.088). Mass-related traits and aspect ratio did not show a correlation with the flight-to-light response (table 2).

**Table 2.** Statistical test results for correlations with flight-to-light response for each morphological trait in *Yponomeuta cagnagella*. Significant *p*-values (< 0.05) are given in bold and statistical trend values (< 0.1) are indicated with a ° symbol. All tests are based on d.f. = 1.

	female		male	
	Wald *χ*^2^	*p*	Wald *χ*^2^	*p*
total body mass	0.69	0.271	0.69	0.451
relative thorax mass	0.00	0.988	0.00	0.974
relative abdomen mass	1.11	0.293	0.22	0.635
wing length	9.92	**0.002**	3.36	0.067°
wing width	3.63	0.057°	1.62	0.219
wing area	9.27	**0**.**002**	3.04	0.081°
aspect ratio	2.19	0.139	0.28	0.599
wing loading	8.61	**0**.**003**	2.23	0.135

Male moths showed qualitatively similar morphological differences: urban males had shorter (by 1.4%), narrower (by 2.5%) and smaller wings (by 3.9%) than rural males (strong evidence) (table 1, figure 2*a–c*). The smaller wing area and the slightly higher body mass in urban males led to higher wing loadings, with strong evidence (table 1). No significant differences were found for relative thorax and abdomen mass and forewing aspect ratio in males either (table 1). Smaller-winged males tended to show a weaker flight-to-light response (table 2, figure 2*f*). This tendency is mainly driven by the difference in wing length between rural and urban moths (tables 1 and 2, figure 2*d,e*). Other morphological traits did not show significant relations with flight-to-light response (table 2).

## 4. Discussion

Urban spindle ermine moths have reduced flight-to-light response compared to rural moths, and it has been hypothesized that such an evolutionary response may be driven by reduced mobility associated with morphological changes [28]. Here, we provide evidence for this reduced mobility hypothesis by contrasting flight-related morphology of urban moth populations that evolved under ALAN conditions and rural, dark-night populations. Urban moths had on average smaller wings, which in turn correlated with a lower probability of being attracted to an artificial light source.

Mobility and dispersal, and associated behaviours, are known to be under strong selection (e.g. [32,33]), because they are associated with foraging, mating and dispersal. Effects of urbanization on mobility-related morphology have been found for some organisms, such as *Anolis* lizards [34,35]. In insects, wing size and aspect ratio are generally considered to play a key role in flight performance and are positively related to acceleration [23] and dispersal capacity [27,36]. High wing loading leads to increased flight and dispersal capacity, but also to decreased manoeuvrability [37]. Therefore, flight-related morphological traits are expected to be under selection. Our findings suggest that urban moths might have reduced acceleration and dispersal capacity compared to rural moths and that urban males may have reduced manoeuvrability due to increased wing loading.

A confounding factor of flight-related traits can be body and thorax size, which often covary positively with wing traits. However, we did not find changes in body size in urban populations. Independence of wing morphology and body size is also supported by a study on the noctuid moth *Agrotis exclamationis*, where body size, but not wing length, increased over 137 years of urbanization [38], possibly leading to higher mobility [26]. However, this study was based on museum voucher specimens, and differences could thus be due to plasticity effects or non-random sampling. Furthermore, selection regimes on flight behaviour may act differently on this generalist species with high mobility.

We acknowledge that other traits may also have been under selection during adaptation to urban environments, for example compound eye morphology and physiology [28,38]. We could not measure eye morphology, yet follow-up studies may address these and other traits in *Y. cagnagella* as well. The moths used in the experiments were collected as larvae in their respective environments. If the traits measured were influenced by maternal effects or very early juvenile experience, the argument for genetic adaptation would need to be re-considered. Two-generation common-garden experiments would allow the effects of maternal phenotype, phenotypic plasticity and genetics to be disentangled.

Habitat fragmentation is an important aspect of current global change and a typical consequence of urbanization. It can trigger both adaptive and non-adaptive evolutionary change [39]. Food specialists, such as *Y. cagnagella,* can adapt to habitat fragmentation by changing their mobility [40]. When the distribution of suitable habitat is highly fragmented, reduced inter-patch dispersal capacity can be associated with higher fitness [41]. This, combined with the negative effects of light pollution may have promoted the evolution of reduced mobility [40]. Modified mobility patterns in urbanized, light-polluted populations may also emerge as by-products of selection on other life-history traits. Although there is evidence that dispersal behaviour can evolve independently of other life-history traits [42], the movement patterns we observed may relate to daily or routine movements rather than dispersal *per se*. Further study is now warranted on the behavioural nature of the flights in both males and females. Reduced mobility would in turn reduce gene flow between populations, making them more vulnerable to the consequences of genetic drift. Besides such effects on (meta-)population viability, monitoring schemes using light traps are affected too [28]: in addition to reduced contrast and hence reduced flight-to-light response in light-polluted areas, moths have species-specific attraction radii [43] that may become smaller following ALAN-driven reduced mobility, further reducing flight-to-light response. This implies that negative population trends obtained from light-trap based monitoring schemes in light-polluted regions could partly be due to physical and evolved trapping bias, yet the extent of this is not known.

As the negative effects of light pollution on the ecology of many species become apparent, it becomes important to understand to what extent organisms may evolve in response to these new conditions. Altermatt & Ebert [28] provided evidence for this by demonstrating a reduced flight-to-light response in urban moths, and we have shown that this is related to changes in wing morphology. The negative effects of light pollution highlight that in urban landscapes we need to strive for green and dark corridors that can functionally connect populations of less mobile species.

## Data Availability

All data and R code used for the analyses are available from the Dryad Digital Repository: https://doi.org/10.5061/dryad.qz612jmp5 [44]. Electronic supplementary material is available online [45].
